# Aspirin and Decreased Colon Cancer Risk: Challenges Interpreting a Large Prospective Trial

**Published:** 2012-11-01

**Authors:** Rita Wickham

**Affiliations:** Independent Oncology and Palliative Care Consultant

It is possible that your patients read the headline, "Aspirin may cut colon cancer risk by 60%," published in *USA Today* in late October 2011, or that they saw a similar account on the Internet. This and other stories appeared the same day results of the CAPP2 trial (Burn et al., 2011) were reported in the medical literature. This study concluded that colorectal cancers (CRC) were significantly less common in individuals with Lynch syndrome—carriers of an inherited defective mismatch repair gene that predisposes them to hereditary nonpolyposis colon cancer (HNPCC) and other cancers—who took aspirin 600 mg per day than would be predicted in a population of people with Lynch syndrome. Oncology advanced practitioners (APs) should assume that some of their patients might interpret this finding to mean that the possible anticancer effect of aspirin may benefit them.

This article will briefly review the potential anticancer effects of aspirin and nondigestible dietary or supplemental fiber (starches) that many of the participants in this trial took. In addition, some strategies for reading and interpreting complex prospective randomized studies such as CAPP2, as well as potential applications and limitations in clinically applying such data, will be discussed.

## Aspirin and Other NSAIDs

The earliest records of people using white willow leaves and bark to reduce fever and musculoskeletal pain date back to the Assyrians in 4000 BC (Mahdi, 2010). In 1874, the principal component of willow (salicin) was identified, and salicylic acid was formulated in the Bayer laboratories in Germany (Vane & Botting, 2003). This rapidly led to Bayer developing a more efficacious and tolerable derivative, acetylsalicylic acid (named Aspirin), which was patented in 1899. After Bayer lost its patent, it became known generically as aspirin (Vonkeman & van de Laar, 2010). Aspirin was available over-the-counter in Germany by 1911, but it remained expensive.

An interesting sidebar: Bayer also synthesized Heroin [after "heroic"] in 1899 and marketed it along with aspirin as both the "healthier" analgesic of the two and as a children’s cough medicine. Tons were sold in Germany and 23 other countries until 1913, when heroin’s addictive potential and numerous hospitalizations became apparent. Bayer took heroin off the market, and aspirin became even more popular.

In 1938, gastric bleeding and inhibition of platelet aggregation were identified as potential adverse effects of aspirin. Gastric ulcers can extend into the small bowel, and large erosions and ulcers were directly visualized by endoscopy in healthy volunteers who had taken aspirin 100 mg a day for 7 to 14 days (Yeomans, 2011). In the late 1940s, increased bleeding was observed in children who chewed aspirin gum after tonsillectomy (Vonkeman & van de Laar, 2010). The physician who observed this hypothesized that aspirin might be effective prophylaxis against cardiovascular events and began to recommend an aspirin a day to his overweight, sedentary, middle-aged male patients and those who had experienced a myocardial infarction. After thus treating thousands of patients, he published his findings in obscure medical journals and proclaimed that aspirin was effective in the prevention of heart attacks and strokes. His recommendations were largely ignored until resurgence of research in this area in the late 1960s, which led to the US Food and Drug Administration (FDA) endorsing low-dose aspirin to decrease the risk of myocardial infarction in the 1980s. The current recommendation for low-dose aspirin use to prevent cardiovascular events is based on noncompetitive, dose-dependent, and irreversible inhibition of cyclooxygenase (COX) on platelets (Gurbel et al., 2007). Other nonsteroidal anti-inflammatory drugs (NSAIDs) competitively and reversibly inhibit COX and do not cause long-term inhibition of platelet aggregation.

The actual mechanism of action of aspirin (and other NSAIDs) was not identified until the 1970s (Vane & Botting, 2003). NSAIDs are "weak" analgesics (compared to the "strong" opioid analgesics) that act in the periphery to reduce nociception and pain by dose-dependent reduction of COX synthesis with resultant inhibition of prostaglandin (PG) formation. By the late 1980s, two COX enzymes were identified: COX-1 and COX-2, to which aspirin and other NSAIDs bind with varying affinities. COX-1 is constitutive, present at all times in low amounts in most tissues, where it acts as a "housekeeping" enzyme to regulate normal processes such as maintaining the integrity of gastric mucosa, supporting normal kidney function, and influencing platelet aggregation. On the other hand, COX-2 is inducible and upregulated after illness or injury. These findings led to the hypothesis that COX-1 is "good" and COX-2 is "bad," and to the development of COX-2–selective NSAIDs, which were proposed to be more effective for pain and inflammation and less likely to cause adverse effects (Vonkeman & van de Laar, 2010).

The true story is far more complex: There are risks for serious adverse effects from COX-2–selective and nonselective NSAIDs. In the US, the only available COX-2–selective NSAID is celecoxib (rofecoxib was removed from the market because of increased risk for myocardial infarction and valdecoxib was removed because of risks for cardiovascular events and severe skin reactions; Ward, Archambault, & Mersfelder, 2010). There are numerous prescription and over-the-counter nonselective NSAIDs, which are classified as salicylates (e.g., aspirin), arylalkanoic acids (e.g., indomethacin, diclofenac, sulindac), profens (e.g., naproxen, ibuprofen, ketoprofen), and others.

The most frequent severe adverse effects of aspirin and other NSAIDs are presented in Table 1 and include gastric (ranging from dyspepsia to serious gastric ulcers with perforation and bleeding), cardiovascular (myocardial infarction, exacerbation of heart failure, and NSAID-induced hypertension), renal (NSAID-induced acute renal failure; Vonkeman & van de Laar, 2010), pulmonary (aspirin-sensitive respiratory disease—also called NSAID hypersensitivity syndrome—and salicylate-induced pulmonary edema; De Weck et al., 2009; Farooque & Lee, 2009; Glisson, Vesa, & Bowling, 2011), and severe skin reactions (Stevens-Johnson syndrome and toxic epidermal necrolysis; Ward et al., 2010).

**Table 1:**
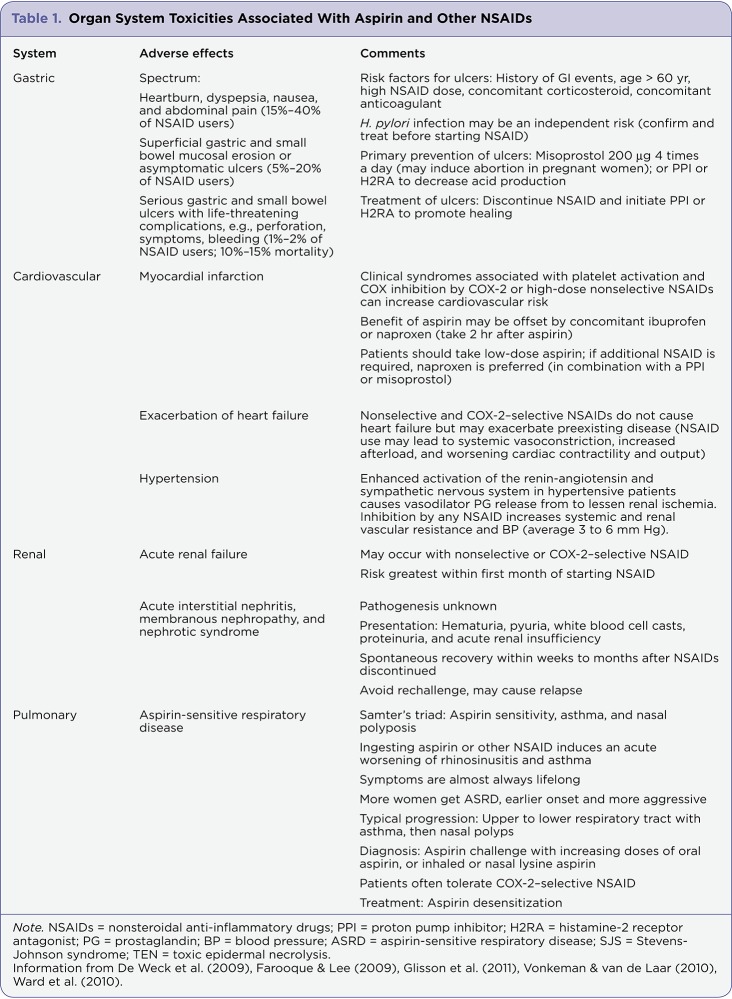
Table 1. Organ System Toxicities Associated With Aspirin and Other NSAIDs

**Table 1:**
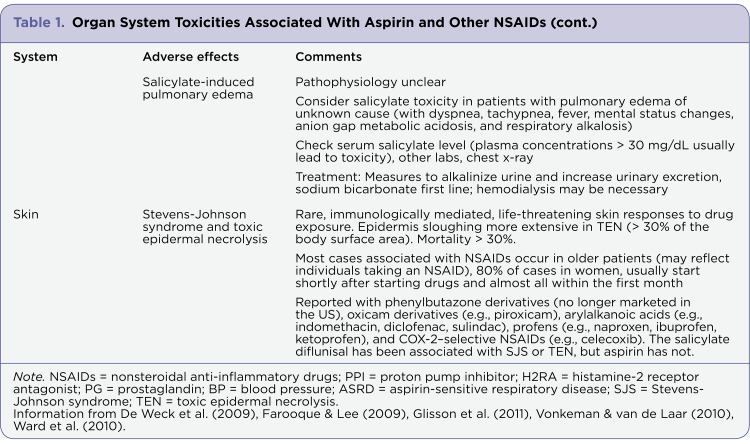
Table 1. Organ System Toxicities Associated With Aspirin and Other NSAIDs (cont.)

It seems counterintuitive that low-dose aspirin is recommended to decrease the risk for myocardial infarction and stroke (because of COX inhibition in platelets), while higher therapeutic doses may cause cardiovascular events. Cardiovascular problems may occur because of reactions to intraluminal blood vessel damage (e.g., from atherosclerotic plaques) that induce platelet production of COX-1–dependent thromboxane A2 that autoactivates platelets and causes vasoconstriction. Damage concomitantly upregulates COX-2 expression and causes PGI2 production that inhibits platelet thrombosis and causes vasodilation. Together, these events alter normal homeostasis in favor of thrombogenesis and vasoconstriction, and NSAID-related COX inhibition (especially COX-2–selective NSAIDs) thereby increases the risk for cardiovascular events (Vonkeman & van de Laar, 2010).

All prescription NSAIDs have black box warnings for increased risk of heart attack, ischemic stroke, gastrointestinal bleeding, and Stevens-Johnson syndrome, which has been linked to the use of certain NSAID painkillers. Before prescribing an NSAID, the AP needs to assess and discuss with each patient the his or her risk for serious adverse effects, particularly gastrointestinal and cardiovascular events.

## Aspirin and Cancer

Interest in the potential relationship between aspirin use and cancer began 30 years ago when it was noted that thrombocytopenic rats with cancer developed fewer metastases than rats with normal platelet levels, and that PG levels were elevated in rat colon cancer tumors. This led to the notions of COX involvement and inhibition in cancer development and progression (Elwood, Gallagher, Duthie, Mur, & Morgan, 2009). In vitro studies have identified potential antineoplastic mechanisms: Aspirin suppresses PG in colon mucosa, inhibits COX-1, has dose-dependent antiproliferative effects, induces cellular apoptosis, and may affect the DNA mismatch system. Subsequent epidemiologic studies have supported an inverse relationship between NSAID (particularly aspirin) use and the incidence of colorectal and breast cancers and perhaps esophageal, gastric, lung, prostate, and skin cancers as well (including melanoma; Cuzick et al., 2009; Johannesdottir et al., 2012). For instance, such studies showed that people who regularly took aspirin had about 40% lower incidence of CRC than those who did not take aspirin (Cuzick et al., 2009).

One large population-based prospective cohort study that included 74,250 Swedish men and women found a significant reduction in the risk for CRC, but only in individuals who took aspirin for at least 20 years (Larsson, Giovannucci, & Wolk, 2006). Similarly, Rothwell and colleagues (2011; 2012) conducted two large analyses of data from patients in randomized trials and examined the differences in cancer deaths and cancer metastases in patients with various cancers (e.g., stomach, colorectal, prostate, esophageal, pancreatic, brain, and lung) who were allocated to taking aspirin vs. not taking aspirin. However, these studies found that daily aspirin taken for 5 years or more was associated with both reduced cancer deaths and delayed metastases.

In another large meta-analysis, Luo and colleagues (2011) examined breast cancer risk and aspirin use in 33 cohort or case-control studies and one randomized prospective study that included 1,916,448 subjects. Pooled analysis of all studies found aspirin use was associated with reduced breast cancer risk (odds ratio = 0.86, 95% confidence interval = 0.81–0.92). These authors concluded that regular aspirin use may be associated with reduced risk of breast cancer, but that randomized controlled trials are needed to confirm the association.

On the other hand, other research did not support a relationship between aspirin use and decreased CRC incidence. The Physicians’ Health Study, which included more than 22,000 male physicians, examined the effect of random assignment to aspirin at 325 mg every other day along with individuals who elected to continue aspirin after it was stopped early. There was no difference in the risk for colorectal or other cancers among individuals who took aspirin and those who did not (randomized and observational analysis), and the authors concluded that low-dose aspirin for 12 years did not reduce the risk for and incidence of cancer (Sturmer et al., 1998). Similarly, the Women’s Health Study included 39,876 women who were randomly assigned to take aspirin at 100 mg or placebo every other day and followed for an average of 10.1 years. There were no observed positive effects of aspirin on breast, colorectal, or other cancers (Cook et al., 2005).

A possible explanation for the differences between these two studies and many of the others is the administration of aspirin every other day instead of every day. Furthermore, the primary purpose of these studies was not to detect CRC occurrence in participants.

The only guideline statement regarding aspirin or other NSAID use for primary prevention of CRC was published by the US Preventive Services Task Force (USPSTF), which recommends against routine use in persons at average risk for CRC (2007). The USPSTF recognizes that aspirin and NSAIDs taken at higher doses and for longer periods reduce colon polyps and perhaps CRC, but this is countered by little evidence for reduced mortality from CRC and the risks for GI bleeding, renal impairment, and possible hemorrhagic stroke. More recently, an international consensus panel stated that the antitumor effect of aspirin and sulindac are "very probable," but that this is only "possible" for other NSAIDs because of the dearth of evidence (Cuzick et al., 2009). This report identified questions that need to be answered, such as the lowest effective dose and duration of NSAID treatment to meaningfully reduce cancer risk, as well as how to identify individuals who would most benefit from treatment.

## Reading the Research Report From Burn and Colleagues

As mentioned, the Burn et al. study report (2011a) is complex and somewhat difficult to read. Clear advantages to the CAPP2 trial were its large size—937 patients were eligible to be randomized to aspirin 600 mg per day or aspirin placebo plus resistant starch 30 g per day or resistant starch placebo—and long-term follow-up. However, another 76 patients who consented to the study refused to take aspirin because of concerns about ulcers, and so were randomized only to resistant starch or starch placebo (they were not included in the analysis). Thus, 861 patients were included in the current analysis, although the authors reported 191 participants had dropped out of the study over time (Burn et al., 2011b).

Patients were randomized in blocks of 16 in a 2 × 2 factorial design (Burn et al., 2011a). A hypothetical example is included in Table 2 and illustrates random assignment to a study comparing aspirin or no aspirin (aspirin placebo) and resistant starch (a supplement of oral indigestible fiber) or no fiber (resistant starch placebo); in all likelihood, the groups would not be exactly the same size. As Table 3 shows, the evaluable group sizes were smaller because of the patients who refused to take aspirin. In addition, 9 patients received aspirin alone and 10 received only aspirin placebo, which must have been an (unexplained) protocol violation. Randomization in blocks is done to try to eliminate the concern of uncontrolled intervening variables (other factors that could increase individual patients’ risk for CRC), which could be problematic because patients were randomized in 43 different international sites. Blocking is also done in an attempt to keep group sizes more equivalent.

**Table 2:**
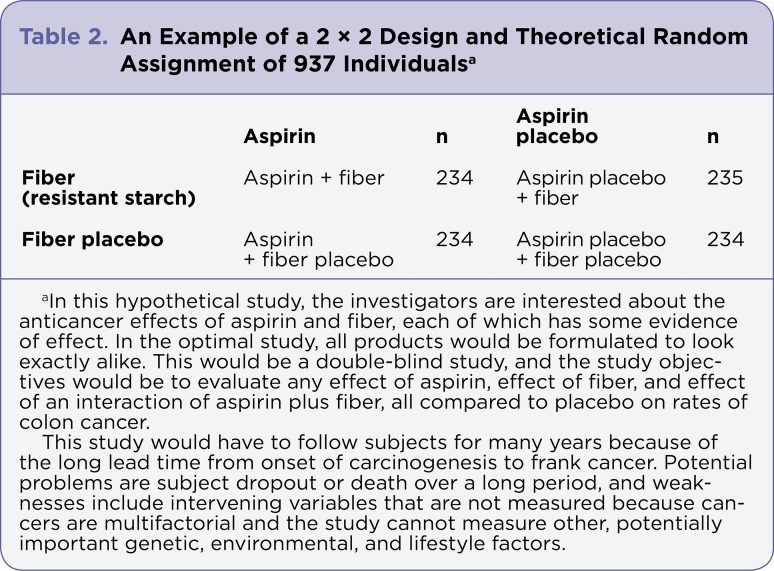
Table 2. An Example of a 2 × 2 Design and Theoretical Random Assignment of 937 Individuals(a)

**Table 3:**
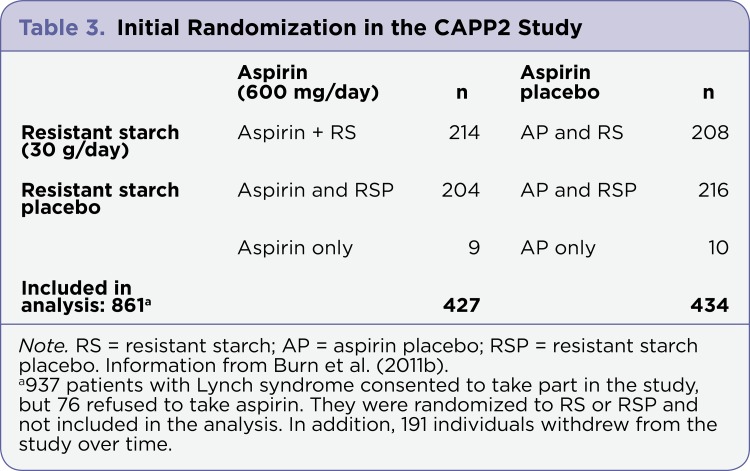
Table 3. Initial Randomization in the CAPP2 Study

In this study, the major variable of interest was the number of colorectal cancers that developed in the patients who took aspirin versus those who did not (Burn et al., 2011a). After a mean of 55.7 months, 18 patients randomized to aspirin and 30 in the aspirin placebo group developed a primary CRC. The authors concluded aspirin 600 mg per day for an average of 25 months substantially reduced CRC risk, a finding that was not statistically significant in the intent-to-treat analysis, but was so in the Poisson regression analysis (*p* = .05), which incorporates more follow-up data than regular regression analysis. In addition, there was no mention of Bonferroni adjustment, the simplest method to correct for multiple comparisons, and no power analysis was done in this study.

It is interesting that any influence or interaction of starch on the effect of aspirin was not specifically measured, though it was purposefully included as a "treatment" and about half of the patients in the study received it (with aspirin or aspirin placebo).

It would be useful to know if reanalysis of the data after longer-term follow-up would come to the same or a different conclusion about resistant starch, particularly in light of the now positive effect of aspirin. This is an important point because low dietary intake of intake of fruits, vegetables, and fiber from other sources (cellulose, pectin, etc.) has been thought to be a risk factor for CRC, and because ecologic studies have shown a possible association between diets low in fiber and high in fat to CRC (Lipkin, Reddy, Newmark, & Lamprecht, 1999; Sellers et al., 1998).

One small study included 25 patients with recent removal of colon adenomas who took rapidly absorbable maize starch each day for 4 weeks (a very short time period). Modest changes were observed in decreased soluble bile acids in fecal water but markers of cell proliferation did not change (Grubben et al., 2001). On the other hand, more sustained ingestion of resistant starch in addition to dietary fiber may have a greater effect. For instance, different types of nonabsorbable fiber were associated with decreased formation of aberrant crypt foci, which may be a precursor to colon cancer, in one animal study (Coleman, Landstrom, Royle, Bird, & McIntosh, 2002). In contrast, other larger observational studies have found negative or inconsistent results for effects of dietary fiber intake and CRC (Park et al., 2005; Schatzkin et al., 2007; Turati et al., 2011).

## Conclusions

Back to the headline: "Aspirin may cut colon cancer risk by 60%." Certainly, this was an overblown headline, but cancer patients typically look for any and all interventions that might help them "conquer" their cancer. When our patients ask us questions about results such as these, we must try to read and interpret the original research reports that do not necessarily give us conclusive answers about CRCs or other primary cancers. CAPP2 may ultimately provide more useful scientific data to add to evidence-based interventions for persons with Lynch syndrome—both in terms of aspirin and resistant starch. Perhaps when the study has matured, the investigators will publish a third article about the study findings. We are also reminded about the complexity of multicausal diseases that develop over time, as well as the influences of genetic inheritance (nature) and environment and lifestyle (nurture).
